# Symmetry of gamma distribution data about the mean after processing with EWMA function

**DOI:** 10.1038/s41598-023-39763-6

**Published:** 2023-09-12

**Authors:** Mohammad M. Hamasha, Mohammed S. Obeidat, Khalid Alzoubi, Ghada Shawaheen, Ahmad Mayyas, Hesham A. Almomani, Akram Al-Sukkar, Adnan Mukkatash

**Affiliations:** 1 Department of Industrial Engineering, Faculty of Engineering, The Hashemite University, P.O.Box 330127, Zarqa, 13133 Jordan; 2 Department of Industrial Engineering, Faculty of Engineering, Jordan University of Science and Technology, P.O. Box 3030, Irbid, 22110 Jordan; 3 Department of Industrial and Systems Engineering, Khalifa University, Abu Dhabi, United Arab Emirates

**Keywords:** Statistics, Engineering

## Abstract

Statistical Process Control (SPC) plays a vital role in maintaining quality and reducing variability in manufacturing processes. Among SPC techniques, the Exponentially Weighted Moving Average (EWMA) stands out for its ability to detect small process shifts quickly, making it a valuable tool in ensuring product consistency and preventing quality issues. EWMA constructs control charts to monitor process mean shifts, tracks product/service quality by identifying variations, and monitors manufacturing process parameters for early detection of deviations and necessary adjustments. EWMA control chart has been proposed as an alternative to the Shewhart control chart. Sequential measurements are processed using the EWMA function before being placed on the control chart. One of the crucial concerns about the EWMA control chart is the asymmetry of the data around the mean. Although processing with the EWMA function reduces data skewness, the problem of asymmetric data may not be solved. The control chart is designed to leave in front of the upper control limit (UCL) α/2 of the data and behind the lower control limit (LCL) another α/2 of the data, and this does not occur in the case of symmetric data. α/2 represents the significance level for each tail in a two-tailed hypothesis test, indicating the probability of incorrectly rejecting the null hypothesis for each side of the distribution. Since many of the distributions in real life can be approximated by the Gamma distribution, the Gamma distribution was adopted in this study. The Monte Carlo simulation methodology was implemented to generate Gamma distributed data, process it with EWMA function and assess the skewness and kurtosis. The purpose of this paper is to evaluate the effect of EWMA parameters on the performance of the EWMA control chart. Moreover, it focuses on skewness and kurtosis reduction after data processing using the EWMA function. The findings help researchers and practitioners to select the best parameters. Further, the research investigates the effect of EWMA parameter on the shape of distribution.

## Introduction

Exponential weighted moving average (EWMA) is recursive function originated by Ref.^1^. There are two main applications for the EWMA, these include the smoothing trend curves (i.e., gold price) and the statistical control chart. The EWMA is a type of moving average. In this type, all measurements are taken into account when calculating the average. What distinguishes this type is the decreasing weight of the measurements, starting from the current measurement to the oldest one. Equation (1) represents the EWMA statistic.

1 $${Z}_{i}=\lambda {X}_{i}+\left(1-\lambda \right){Z}_{i-1}; \quad i=\mathrm{1,2}, \dots ,n; \; 0\le \lambda \le 1,$$

where ${X}_{i}$ is the current measurement value, ${Z}_{i}$ is the current EWMA statistic, and ${Z}_{i-1}$ is the EWMA statistic observed at the time of the previous measurement. Weighting factor (lambda, λ) controls the share of the last measurement in the value of the EWMA statistic. Thus, it affects all shares of previous measurements depending on how far they are from the current measurement. For example, if λ = 0.6, and the current measurement is the fourth, then the weight of the current measurement (i = 4) is 0.6, the weight of the previous measurement (i = 3) is 0.24, and the weight of the measurement at i = 2 is 0.096. The rest of the weight is for the first measurement (i = 1), which is 0.064. If the number of measurements is relatively large, then the effect of the old measurements becomes very small, especially if lambda is large. If the lambda equals 1, then the EWMA takes its value from only the most recent measurement.

### The EWMA chart

To ensure that the industrial and service operations continue at the same pace without deviation, quality engineers must monitor appropriate process measurements and ensure that they do not deviate. The process is usually controlled by statistical process control charts. Inaccuracies in determining appropriate measurements or control systems may extensively affect product quality and process efficiency. Statistical process control is an important method of monitoring and controlling processes by highlighting the presence of any deviation affecting products. EWMA is important in statistical process control (SPC) because it provides a dynamic and responsive method for monitoring process variations. By assigning exponentially decreasing weights to observations, EWMA emphasizes recent data while considering historical information, enabling timely detection of shifts or trends in the process. This allows for prompt corrective actions, improving product quality and overall process performance.

The Shewhart control chart differs from the EWMA chart in term of values plotted on the chart. Shewart judges the process to be out of control when the last point is out of control, and this point represents the last measurement calculated in the last sample. However, judging the process out of control in the EWMA control chart is based on an exponential weighted average that includes all past measurements and the current one. By choosing an appropriate lambda, the EWMA control procedure could be set to be sensitive to small or incremental deviation in the process^2–7^.

The selection of the lambda parameter in EWMA control charts plays a critical role in determining the chart's sensitivity to detecting process shifts. Several studies and researchers have provided recommendations on the appropriate range of lambda values, contributing to the variation in suggested values for optimal outcomes. One such recommendation mentioned by the authors is provided by Hunter^8^, suggesting a range of [0.2, 0.3] for lambda. Hunter's recommendation emphasizes the importance of considering the width of the control region, denoted as L, in combination with lambda to achieve the desired in-control Average Run Length (ARL). On the other hand, Montgomery^9^ recommends lambda values between 0.05 and 0.25. Montgomery's recommendation is based on extensive research and aims to optimize the performance of EWMA control charts. The rationale behind these varying recommendations stems from the trade-off between chart sensitivity and false alarms. Higher lambda values increase chart sensitivity, making it more responsive to smaller shifts in the process mean. However, this may also lead to an increased risk of false alarms, triggering unnecessary investigations and interventions. Conversely, lower lambda values reduce false alarms but may result in decreased sensitivity to detect smaller shifts. The choice of lambda should, therefore, consider the specific context, desired level of sensitivity, and acceptable false alarm rate. It is crucial to recognize that lambda alone does not solely determine the chart's sensitivity; it must be considered in conjunction with the width of the control region, L. By incorporating these considerations, researchers and practitioners can make informed decisions when selecting lambda values, ensuring the effectiveness of EWMA control charts in monitoring and improving process performance.

Many researchers have focuses on EWMA control chart from different sides. The following are examples about very resent research: Noor et al.^10^ introduced a hybrid exponentially weighted moving average (HEWMA) control chart based on a Bayesian approach. The chart incorporated two different loss functions (symmetric and asymmetric) and considered informative and non-informative priors. Performance evaluation of the proposed chart utilized the average run length (ARL) and standard deviation of run length (SDRL) measures. Extensive simulations were conducted to assess its performance, and a real data example demonstrated its implementation. Anwar et al.^11^ proposed an auxiliary information-based double homogeneously weighted moving average (DHWMA) control chart for monitoring process location. The chart utilized auxiliary information from a CUSUM chart to enhance its performance. ARL and SDRL were used as performance measures, and the study included simulations and a real data example. Chong et al.^12^ focused on optimizing the exponentially weighted moving average (EWMA) median chart by considering the median run length (MRL) as a performance measure. They used the expected median run length (EMRL) as an objective function for optimization and provided an illustrative example to showcase the application of the optimal EWMA median chart. In a separate study, Chatterjee et al.^13^ introduced the maximum double generally weighted moving average (Max-DGWMA) chart, which detects shifts in process mean and/or process dispersion. Through Monte Carlo simulations with time-varying control limits, the Max-DGWMA chart's run length performance was compared to other charts, demonstrating its superior efficiency compared to the Max-EWMA, Max-DEWMA, and Max-GWMA charts, while being comparable to the SS-DGWMA chart. An application in the automotive industry illustrated the implementation of the Max-DGWMA chart. Abbas et al.^14^ introduced a new mixed EWMA-progressive mean (MEP) chart, which combines robustness and sensitivity for process monitoring. Abbas et al.^15^ introduced the nonparametric progressive mean sign (NPPM-SN) chart for monitoring process targets in manufacturing and service production. It compares the NPPM-SN chart with other control charts using Monte Carlo simulation, finding it more robust and efficient, particularly for skewed distributions. Real-life and simulated examples are provided.

The upper control limit (UCL) and lower control limit (LCL) in EWMA depend on the number of previous measurements (i.e., *i*), as shown in Eqs. (2) and (3).

2 $$UC{L}_{i}={\mu }_{0}+L\sigma \sqrt{\left(\frac{\lambda }{2-\lambda }\right)\left[1-{\left(1-\lambda \right)}^{2i}\right]},$$

3 $$LC{L}_{i}={\mu }_{0}-L\sigma \sqrt{\left(\frac{\lambda }{2-\lambda }\right)\left[1-{\left(1-\lambda \right)}^{2i}\right]}.$$

However, at very large value of *i,* the component ${\left(1-\uplambda \right)}^{2\mathrm{i}}$ becomes very close to zero, and both the UCL and LCL equations might be reformed as expressed in Eqs. (4) and (5).

4 $$UCL={\mu }_{0}+L\sigma \sqrt{\left(\frac{\lambda }{2-\lambda }\right)},$$

5 $$UCL={\mu }_{0}-L\sigma \sqrt{\left(\frac{\lambda }{2-\lambda }\right)}.$$

A lower value of lambda leads to a lower current measurement weight and then a smoother trend. Hunter^8^ recommended lambda to be 0.2–0.3. L can be chosen to control the average run length (ARL) to locate a false signal (e.g., ARL = 370 or 500). Borror et al.^16^ and Lucas and Saccucci^17^, for example, worked on L selection.

### Gamma distribution

The Gamma distribution of non-negative real numbers is known, and it is a continuous distribution with two positive parameters, scale and shape parameters. Here, the scale parameter is the average probability of the event under consideration for each time period. However, the shape parameter considers its change over time. This means that the gamma distribution can be flexibly adapted to experimental conditions and used in a variety of areas, such as designing insurance conditions^18^, modeling genetic fingerprints^19^, and calculating the required volume of water reservoirs during rainfall^20^. Density and cumulative density functions of the Gamma distribution are formulated with the Gamma function. Equation (6) represents gamma function, and Eq. (7) is a special characteristic of Gamma function.

6 $$\Gamma \left(x\right)={\int }_{0}^{\infty }{t}^{x-1}.{e}^{-t}dt, \quad \mathrm{ for } \; x>0,$$

7 $$\Gamma \left(x+1\right)=x.\Gamma (\mathrm{x}) \; \mathrm{ and \; \Gamma }\left(1\right)=1.$$

Gamma-distributed random variable X has a density function as addressed in Eq. (8) along with a cumulative density function as addressed in Eq. (9).

8 $$f\left(x;\theta ,k\right)=\frac{{x}^{k-1}.{e}^{-x/\theta }}{{\theta }^{k}.\Gamma \left(\mathrm{k}\right)}, \quad \mathrm{ for \; all } \; x,\theta ,k>0,$$

9 $$F\left(x;\theta ,k\right)={\int }_{0}^{x}\frac{{u}^{k-1}.{e}^{-u/\theta }}{{\theta }^{k}.\Gamma \left(\mathrm{k}\right)} du, \quad \mathrm{ for \; all } \; x,\theta ,k>0.$$

**Figure 1:**
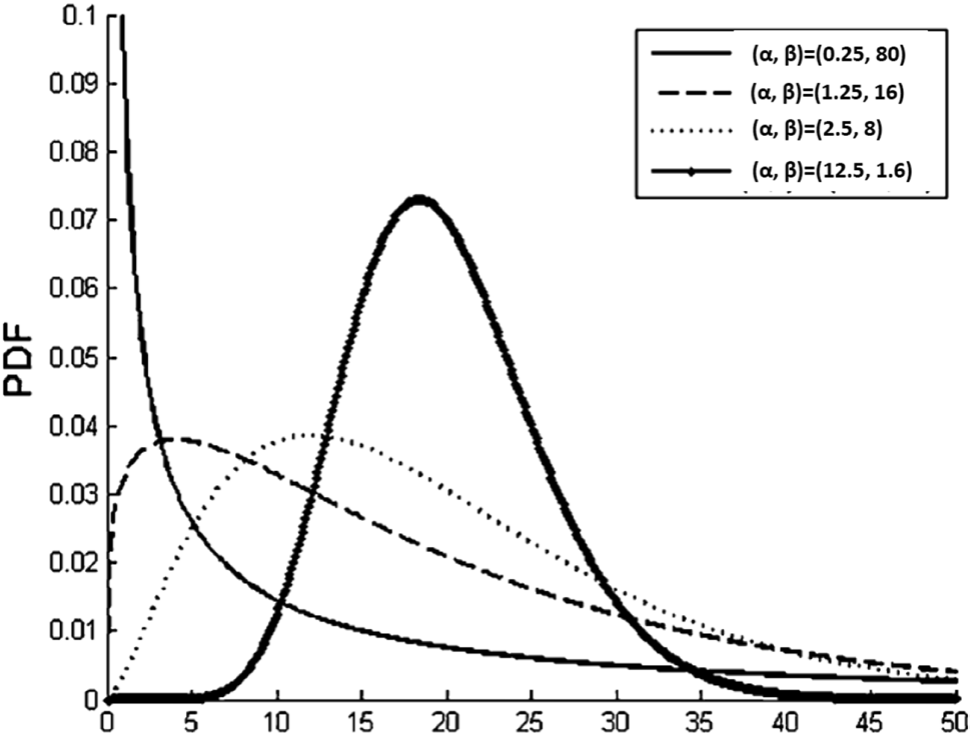
Some shape of the Gamma distribution^22^.

The Gamma distribution could be very skewed to the right or very symmetric about its center, which is a reasonable approximation of most probability distributions with positive measures. Figure 1 shows the shape of a few Gamma distributions. The exponential distribution is the most gamma skewed state and this particular case could be achieved when the value of $\theta$ is 1. Moreover, at $\theta \to \infty$, Gamma distribution becomes symmetric around the mean, and reasonably approximated into a t-distribution^21^. A real life distribution with any degree of skewness to the right can be approximated with gamma distribution by selecting optimum values for $\theta$ and k. Even if the distribution is skewed to the right, it can be approximated using the gamma reflected about its mean. Most software tools (such as MS-Excel) use alpha instead of θ and beta instead of k.

The Gamma distribution is highly popular and widely utilized in research and various applications. The term "Gamma Distribution" has been searched over 4 million times on Google Scholar, with thousands of searches conducted since July 11, 2023. Numerous studies conducted in the current year have predominantly focused on the theoretical aspects of the Gamma distribution^23–29^.

In details, several recent studies have contributed to the understanding and application of the gamma distribution in various fields. Aslam et al., proposed a control chart for gamma distributed quality characteristic, using gamma to normal transformation and multiple dependent state sampling. It outperforms the Shewhart chart, as shown in parameter analysis and a real data case study. Chen et al.^24^ developed a comprehensive toolbox, known as the gammadist package, to enhance the analysis of the gamma distribution. Al-Omari and Dobbah^25^ investigated the mixture of Shanker and gamma distributions and its relevance to engineering data. Rao and Aslam^26^ proposed different sampling inspection plans for cancer patients based on the gamma distribution. Wilson et al.^27^ introduced the compound variance gamma distribution for modeling randomly scattered signals and detection using two sensors. Ilinca and Anghel^28^ applied the gamma family probability distributions to flood frequency analysis. Finally, Adam et al.^29^ developed a generalized gamma distribution for biomedical signals denoising.

Furthermore, there are numerous studies in the current year that have focused on the application aspects of the Gamma distribution^30–38^. In details, the extreme values of two probability functions for the Gamma distribution were investigated by Sun et al.^34^ in their study published in the Journal of Scientific Reports. Nawa and Nadarajah^35^ proposed new closed form estimators for a bivariate gamma distribution in the journal Statistics. In another study published in the Journal of Scientific Reports, Sun et al.^34^ again examined the extreme values of two probability functions for the Gamma distribution. Edelmann et al.^36^ derived product inequalities for multivariate Gaussian, gamma, and positively upper orthant dependent distributions in the journal Statistics and Probability Letters. Masmoudi and Rejeb^37^ explored the asymptotic behavior and parameter estimation of the infinitely divisible matrix gamma distribution, which was published in Statistics and Probability Letters. Nascimento et al.^38^ proposed a compound truncated Poisson gamma distribution to understand multimodal SAR intensities in the Journal of Applied Statistics.

Since the gamma distribution is flexible to approximate wide range of real-life distributions, it was chosen to study the effect of the EWMA function on skewness and kurtosis.

The gamma distribution is chosen to study the effect of parameters on skewness and kurtosis of EWMA statistics for the following reason. As a versatile distribution, the gamma distribution can be used to simulate a variety of wait durations. Using maximum likelihood estimate, fitting to data is also not too difficult. The fact that the gamma distribution belongs to the exponential family of distributions is one of the many characteristics that make it helpful for statistical analysis. The gamma distribution can be used in a variety of studies, including timing the amount of time it takes for a website to load, a phone call to be answered, or a radioactive atom to decay. Other experiments include measuring how long it takes to service customers at checkout counters.

The wait time until a predetermined number of events has occurred can be modeled using the gamma distribution in each of these scenarios. This can be helpful in understanding how various variables, such as the volume of website traffic or the number of consumers in line, affect the wait time. Of course, there are other distributions that can be used to simulate wait times besides the gamma distribution. The exponential distribution, the Weibull distribution, and the log-normal distribution are further possible distributions. The exact characteristics of the data and the experiment's objectives will determine which distribution should be used.

### Effect of data skewness on control chart performance

In this section, effect of skewness on the performance of control chart is discussed. The standard control chart is usually designed by setting the UCL at a distance Lσ above the target, and the LCL at the same distance below the target. The performance of the control chart is ideal if the input distribution to the control chart is symmetric around the mean, where the chance of getting a point higher than the UCL is the similar to getting a point lower than the LCL.

For this reason, Shewhart control chart utilizes central limit theorem. In other words, the means of the subgroups are taken as inputs to the Shewhart control chart, and individual units are only allowed to be used if the distribution of the original data is normal.

EWMA control chart has a similar distance between UCL-target and target-LCL. Therefore, the symmetry around the mean of Z, i.e., EWMA output data, is important to keep a good performance of the control chart. Borror et al.^16^, Stoumbos and Sullivan^39^, and Maravelakis et al.^40^ discussed the robustness to normality of the EWMA, to evaluate the effect of non-normal distribution on the performance of EWMA control chart.

### Novelty and gap of the research

The novelty of this research is that it investigates the effect of EWMA parameters on the performance of the EWMA control chart for asymmetric data. This research will provide new insights into the use of EWMA charts for real-world data that is often asymmetric. The research also investigates the effect of EWMA parameters on the skewness and kurtosis of the data. This is important because skewness and kurtosis can affect the performance of control charts, so it is important to understand how EWMA parameters can influence these measures. The research uses Monte Carlo simulation to generate Gamma distributed data, which is a common distribution in real-world applications. This allows the research to simulate realistic data and assess the performance of the EWMA control chart under a variety of conditions. The research findings will help researchers and practitioners to select the best parameters for EWMA control charts. This will improve the ability of these charts to detect process changes and ensure that they are effective for monitoring asymmetric data.

Most previous research on EWMA control charts has focused on symmetric data. However, many real-world data sets are asymmetric, and this can affect the performance of the EWMA control chart. There is a gap in the research on the effect of EWMA parameters on the performance of the EWMA control chart for asymmetric data. This gap needs to be filled in order to understand how to use EWMA charts effectively for monitoring asymmetric data. The findings of this study will contribute to the field of statistical quality control by providing new insights into the use of EWMA charts for real-world data that is often asymmetric. The findings will also help researchers and practitioners to select the best parameters for EWMA control charts and improve the ability of these charts to detect process changes.

## Experimental methodology

In this study, the researchers opted to utilize the Monte Carlo simulation methodology as a means to generate random variates from the Gamma distribution. By employing this simulation technique, a large number of random variates were generated, closely approximating the Gamma distribution. These generated random variates served as the basis for calculating the Exponentially Weighted Moving Average (EWMA) statistics, which were subsequently utilized as the output data for further analysis. By employing the Monte Carlo simulation and leveraging the generated random variates, the researchers were able to assess the performance and behavior of the EWMA statistics, providing valuable insights into the underlying system or process being studied. The Monte Carlo simulation, also known as the Monte Carlo method or multiple probability simulation, is a mathematical technique that provides a method of estimating the result of complex functions by simulating several random directions of the risky factors. The Monte Carlo method was invented by John von Neumann and Stanislaw Ulam during World War II to improve decision making under uncertain conditions (Reference if you have one here). The Monte Carlo simulation obtained its name from a well-known casino city, called Monaco, since the element of randomness is the core of the modeling, similar approach and a game of roulette.

**Figure 2:**
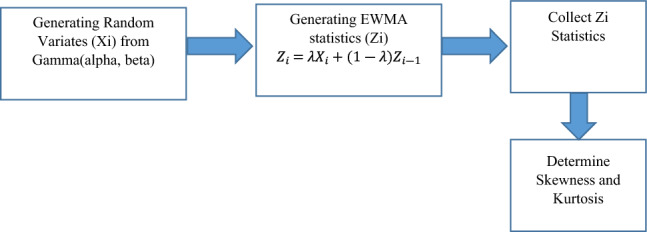
The simulation three steps.

As shown in Fig. 2, the simulation used has three steps, as follow. First, a series of random variates is generated from a given Gamma distribution. Second, the generated random variates are then used to generate a series of EWMA statistics. Pulling random variates when generating EWMA statistics is in the same order as the original series. Finally, the EWMA statistics are collected and analyzed, and both skewness and kurtosis are calculated.

The first computed EWMA statistic (i.e., Z_1_) takes the same value as the resulting variate from the gamma distribution (i.e., X_1_). Then Z_1_ and the second generated random variate, X_2_, are used to calculate Z_2_ as explained in Eq. (1). The process continues until the last random variate is generated. The importance of this figure is to explain the mathematical part of the EWMA estimate along with the simulation steps. The simulation setting is clarified in Table 1.

**Table 1:** Simulation setting.

Simulation setting	Value
Sample size	1000
Distribution	Gamma
Shape parameter (k)	20
Scale parameter	5
Replications	100
Confidence level	0.95
Statistical test	Two-sample t-test
Alpha level	0.05
Power calculation	Power of 0.8
Effect size	Cohen's d = 0.5
Statistical software (execution)	EXCEL
Statistical software (validation)	MATLAB

**Figure 3:**
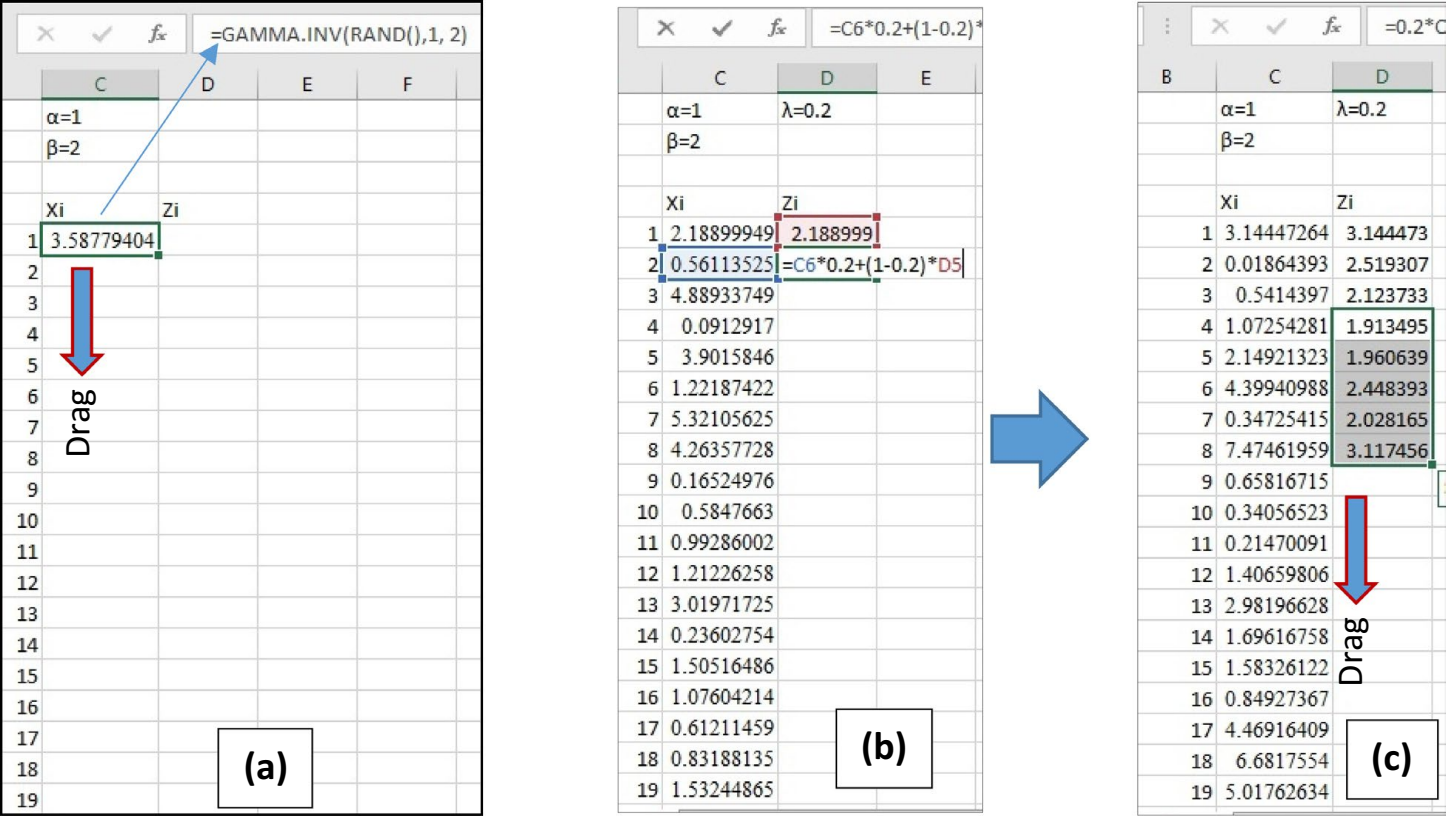
Performing Monte Carlo simulation using MS-Excel in details.

MS-Excel is used to apply Monte Carlo simulation in this paper. Random variables were generated using MS-Excel from the Gamma distribution, as follow. The excel code "GAMMA.INV (probability; alpha; beta)" was used to determine the inverse value of the cumulative density of the Gamma distribution. Continuing to supply and feed random probabilities into the code, "GAMMA.INV(probability; alph;, beta)" will generate random variates from the assigned Gamma distribution. As it is the nature of the probabilities in that they are distributed over the field [0:1], and to obtain non-disordered results, the probability distribution must be uniform. Therefore, the Excel code “Rand()” is used to generate these uniformly distributed random probabilities over the domain [0:1]. Feeding random probabilities into the Gamma inverse code can be achieved by adding “Rand()” to the location of ‘probability’. For example, ‘GAMMA.INV(Rand(); 1; 2)’ if the alpha = 1 and beta = 2. See Fig. 3a. Assigning numbers from 1 to 1 million in the first column helps a lot. Then a random variate from Gamma(1, 2) distribution is generated at the first cell at the top of the column. The next step is either to drag the cell to generate 1 million variates in a column, or just double click on the lower right corner of the cell. The result is a column of 1 million cells filled with random variates fitted to Gamma(1, 2).

After generating a column of random variates, the second step is to generate EWMA statistics in the next column. The first cell at the top of this column takes the same value at the top of random variate column (i.e., 2.188999), and this is performed only for one time. Assuming the lamda is 0.2, the EWMA equation is applied in the second cell with the designation of cell X_2_ (the second cell in the column) and Z1 (the first cell in the column Zi), as shown in Fig. 3b. This cell is dragged or double-clicked in the lower right corner of the cell. One million EWMA statistics were obtained, in other words, inputs to the EWMA control chart, as shown in the Fig. 3c. Further statistical analysis is performed on the EWMA statistics, as the third and last step.

The model was then verified by the authors and validated. For each simulation run, the mean, variance, and skewness of the generated variables were estimated, and compared with theoretical values, as shown in Appendix 1. Further, the error fraction was estimated for every simulation run, as addressed in Eq. (10).

10 $$Error \; Fraction=\left|\frac{Theoritical \; value-Simulation \; value}{Theoritical \; value}\right|.$$

Among all 144 simulation runs, the fraction error did not exceed 0.01 in either the mean or the variance. As for the skewness, in contrast, the error fraction reached 0.02 for one run only, and very few reached 0.01. This was a very good validation of random variates entered for the EWMA function. Hence, the EWMA equation is simple, and there is no chance of error in its application, the EWMA statistics must be accurate since the input variates are validated. For further validation of the result, MATLAB software was used to produce results and compare it to the result. We made sure that our result and MATLAB results are very close. The Matlab code is provided in the Appendix 2.

## Results and discussion

**Figure 4:**
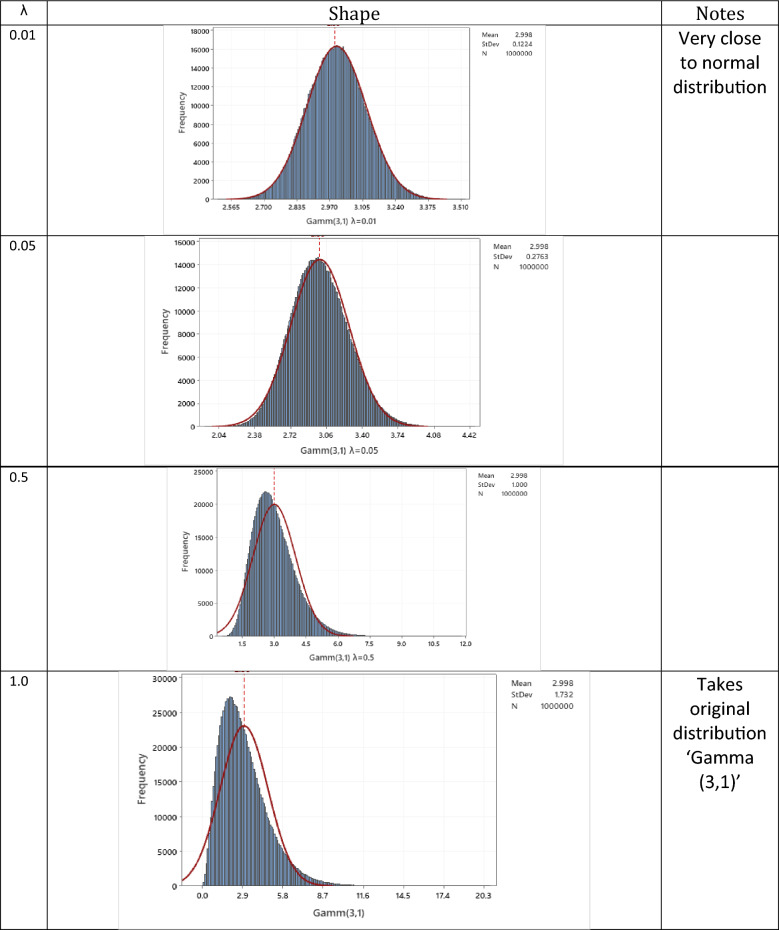
Effect of λ of the shape of EWMA statistics shape for input of Gamma (3,1) distributed.

After generating a million random variates from Gamma distribution, a million EWMA statistics were generated with a certain λ each time. The following values of λ are considered in this study: 0.01, 0.05, 0.1, 0.2, 0.3, 0.4, 0.5, 0.6, 0.7, 0.8, 0.9, and 1.0. The effect of λ on the shape of gamma distribution is quickly. To cover the entire range of lambda uniformly, several values of lambda were selected. These values were chosen to ensure that lambda spans the entire range from 0 to 1 in a uniform manner. This approach allows for a comprehensive exploration of the effects of different lambda values on the study's variables or parameters. By systematically varying lambda across the entire range, researchers can gain a more comprehensive understanding of the relationship between lambda and the phenomenon under investigation. Histogram of EWMA statistics determined from generated 1 million random variate from Gamma (3,1) is plotted vs normal distribution for different value of λ. See Fig. 4. With the decrease in the value of λ, the EWMA statistics distribution shifts gradually from the original gamma distribution to a normal distribution.

The skewness and kurtosis were then estimated for the EWMA statistics. Skewness, or skewness coefficient, or asymmetry coefficient, in descriptive statistics and probability theory is an indicator for measuring the degree and direction of asymmetry of the probability distribution function of a true random variable. In addition to the kurtosis coefficient, it is considered one of the most important formal features of the probability distribution, which enables, along with the parameters of central tendency and statistical dispersion, to understand the structure of variables and statistical data. If the asymmetry is skewed to the right, then the coefficient is negative and positive in the case of a left focused distribution function. In the case of symmetry (as in the case of a normal distribution, this coefficient is zero).

The skewness of the Gamma distribution is a function of a shape parameter that takes only the form 2/$\surd$α. Therefore, the effect of the scale parameter (β) on the deflection after processing with the EWMA function should be zero. In other words, the skewness of the EWMA statistics is the same if the variables come from Gamma (1, 1) and Gamma (1, 10).

**Figure 5:**
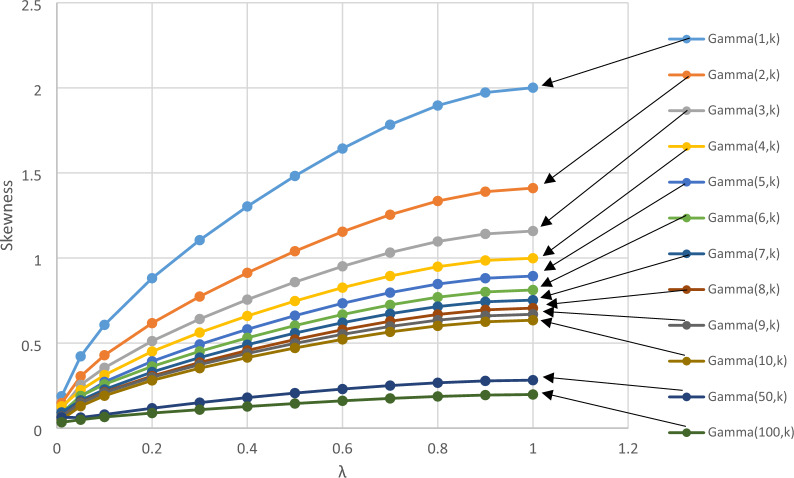
The relationship between skewness and λ after processing EWMA function.

Figure 5 shows a full result about the relationship between skewness and λ, when applying EWMA. Each curve represents a gamma distribution at certain shape parameter, specifically the following: Gamma (1, β), Gamma (2, β), Gamma (3, β), Gamma (4, β), Gamma (5, β), Gamma (6, β), Gamma (7, β), Gamma (8, β), Gamma (9, β), Gamma (10, β), Gamma (50, β), and Gamma (100, β). The curves were very smooth. The skewness for all distributions decreased with decreasing the value of λ. The most skewed distribution considered in Fig. 5 is Gamma (1, β). With decreasing of λ from 1 to 0.01, the skewness was dropped sharply from about 2 to 0.1874758. The lower considered skewed distribution was Gamma (100, β), with original skewness of 0.1976 (skewness of 0.2 theoretically). The drop in the skewness was slight from 0.197573 at λ = 1.0 into 0.0343 at λ = 0.01. Thus, the skewness reduction when decreasing λ is higher for more skewed distribution. Even though, the skewness at λ = 0.01 for Gamma (1, β) was still six folds of skewness of Gamma (100, β) after processing.

Based on the previous discussion, the recommendation is to use low value of λ if the used data distribution is unknown. In this way, the effect on highly skewed data could be reduced.

Kurtosis, also called kurtosis coefficient, flatness coefficient, degree of curvature, or curtosis, is an indicator for measuring the degree of curvature or curvature of the probability distribution function of a true random variable. Similar to skewness, it is one of the most important features of the shapes of random variables’ distributions, and it enabled us to describe the shape of the probability distribution next to the expected value.

**Figure 6:**
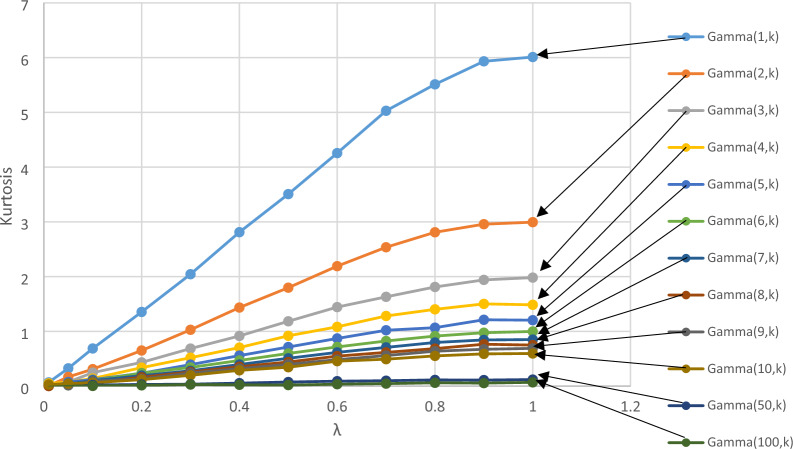
The relationship between kurtosis and λ after processing EWMA function.

Similarly, to the skewness case, kurtosis of the Gamma distribution is a function of a shape parameter, which takes only the form 6/α. Therefore, the effect of the scale parameter (β) on the deflection after processing with the EWMA function is zero. Figure 6 shows a full result about the relationship between kurtosis and λ when applying the EWMA. The same notices about the trends and relationship discussed in skewness case were exactly noticed in kurtosis. The only difference note was that the increment of the curve, which started linear, then become convex while the deflection curves started to be convex from the beginning.

## Case study and sensitivity analysis

The retail shop chosen for this study is a well-known clothing store situated in the bustling city of Amman, Jordan. Located in a vibrant shopping district, the store caters to a diverse range of customers, including both locals and tourists. Renowned for its fashionable offerings and exceptional customer service, the shop witnesses a significant influx of customers throughout the day. In order to gather data for this study on customer arrival times, a systematic data collection process was implemented. Trained observers were discreetly stationed within the store over a span of several weeks to record the precise arrival times of customers. The process of data collection entailed capturing the arrivals of customers from the moment they entered the store, thus ensuring a comprehensive representation of customer arrival patterns. Through the collection of real-time arrival data, the objective is to analyze and comprehend the temporal dynamics of customer flows.

The optimization of operations and the enhancement of customer service in retail stores heavily rely on the comprehension of customer arrival patterns. In this case study, emphasis is placed on the analysis of customer arrival time within a retail store situated in Amman, the vibrant capital city of Jordan. Through the utilization of empirical observation and data analysis, it was ascertained that the arrival time of customers in this particular store exhibits characteristics that are in line with the gamma distribution. The retail sector in Amman reflects a dynamic market influenced by cultural practices and consumer behavior specific to the city. By investigating customer arrival times within a retail store in this context, valuable insights are obtained concerning the temporal dynamics of customer flows. This, in turn, aids in operational planning and resource allocation. Throughout the study, data on customer arrival times at the store were collected over a specified period. It was observed that the distribution of arrival times displayed discernible patterns that closely corresponded to the gamma distribution, which is commonly utilized for modeling inter-arrival times. The gamma distribution offers a flexible framework for describing the variability and interdependency of customer arrival times, enabling a comprehensive analysis and understanding of the underlying arrival processes in the retail store. Since the store commences operations at 9:00 a.m., the first element of arrival times is set at that time. Table 2 presents the inter-arrival times for customers.

**Table 2:** Arrival time and inter arrival time of customers in store.

Arrival no	Arrival time	Inter-arrival time (min)
1	9:09 a.m.	9.6
2	9:14 a.m.	5.1
3	9:17 a.m.	2.5
4	9:20 a.m.	3.5
5	9:26 a.m.	5.9
6	9:28 a.m.	2.4
7	9:33 a.m.	5.4
8	9:36 a.m.	2.7
9	9:48 a.m.	12.4
10	9:59 a.m.	11.3
11	10:09 a.m.	9.9
12	10:17 a.m.	8.4
13	10:23 a.m.	5.8
14	10:25 a.m.	1.8
15	10:35 a.m.	10.4
16	10:49 a.m.	14.0
17	10:55 a.m.	6.0
18	10:59 a.m.	4.4
19	11:10 a.m.	11.2
20	11:22 a.m.	12.0

This case study was conducted with the intention of enhancing the comprehension of customer behavior and arrival patterns within the retail environment in Amman. By establishing the adherence of customer arrival times to the gamma distribution, significant insights were furnished to retail managers and decision-makers operating in Amman's retail industry. These insights possess the capacity to guide strategies for staffing optimization, queue management enhancement, and the overall improvement of customer service, all based on a more profound comprehension of the characteristics associated with arrival times. With conduction sensitivity analyzing by varying the value of λ, the performance of EWMA can be estimated. See Table 3.

**Table 3:** Inter arrival time after applying EWMA with different λ.

	λ										
	0.9	0.8	0.7	0.6	0.5	0.4	0.3	0.2	0.1	0.05	0.01
Inter-arrival time											
9.6	9.6	9.6	9.6	9.6	9.6	9.6	9.6	9.6	9.6	9.6	9.6
5.1	5.6	6	6.5	6.9	7.4	7.8	8.3	8.7	9.2	9.4	9.6
2.5	2.8	3.2	3.7	4.3	4.9	5.7	6.5	7.5	8.5	9	9.5
3.5	3.5	3.5	3.6	3.8	4.2	4.8	5.6	6.7	8	8.8	9.4
5.9	5.7	5.4	5.2	5.1	5.1	5.3	5.7	6.5	7.8	8.6	9.4
2.4	2.8	3	3.3	3.5	3.8	4.1	4.7	5.7	7.3	8.3	9.3
5.4	5.1	4.9	4.8	4.6	4.6	4.6	4.9	5.6	7.1	8.2	9.3
2.7	2.9	3.1	3.3	3.5	3.6	3.9	4.3	5.1	6.6	7.9	9.2
12.4	11.5	10.6	9.7	8.8	8	7.3	6.7	6.5	7.2	8.1	9.3
11.3	11.3	11.1	10.8	10.3	9.6	8.9	8.1	7.5	7.6	8.3	9.3
9.9	10	10.1	10.1	10	9.7	9.3	8.6	8	7.8	8.4	9.3
8.4	8.5	8.7	8.9	9	9.1	8.9	8.5	8	7.9	8.4	9.3
5.8	6.1	6.4	6.7	7.1	7.4	7.7	7.7	7.6	7.7	8.2	9.2
1.8	2.3	2.7	3.3	3.9	4.6	5.3	6	6.4	7.1	7.9	9.2
10.4	9.5	8.8	8.2	7.8	7.5	7.3	7.3	7.2	7.4	8	9.2
14	13.5	12.9	12.3	11.5	10.7	10	9.3	8.6	8.1	8.3	9.2
6	6.8	7.4	7.9	8.2	8.4	8.4	8.3	8.1	7.9	8.2	9.2
4.4	4.7	5	5.5	5.9	6.4	6.8	7.1	7.3	7.5	8	9.1
11.2	10.6	10	9.5	9.1	8.8	8.6	8.4	8.1	7.9	8.2	9.2
12	11.9	11.6	11.2	10.8	10.4	9.9	9.5	8.9	8.3	8.4	9.2

**Figure 7:**
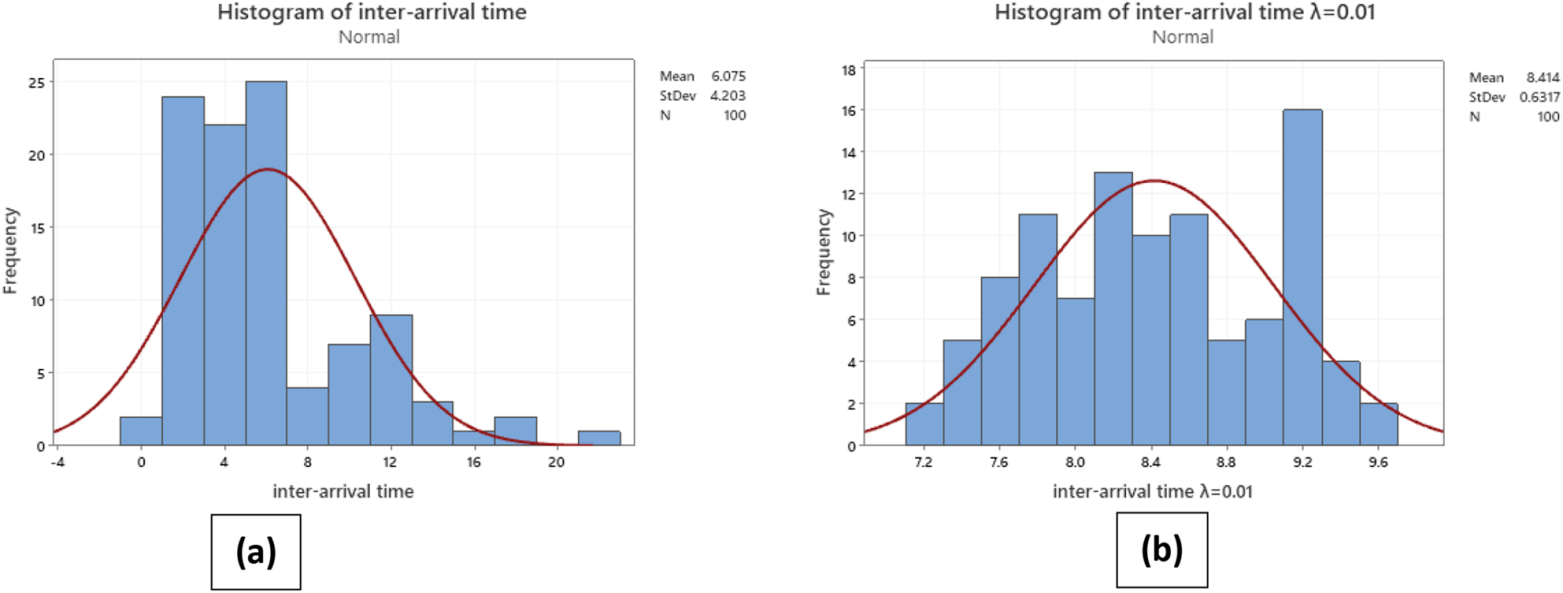
(**a**) Inter arrival time before applying EWMA. (**b**) Inter arrival time after applying EWMA λ = 0.01.

Upon the implementation of the EWMA function to the customer arrival time data within this case study, significant changes and improvements were observed. The utilization of EWMA yielded a smoothed depiction of the arrival time series, effectively diminishing the impact of noise and random fluctuations. The smoothed data offered a clearer perspective on the underlying trends and patterns in customer arrival, thereby facilitating a more comprehensive understanding of the temporal dynamics involved. Furthermore, through the allocation of higher weights to recent data points, the EWMA function enabled real-time detection of alterations in arrival patterns. Anomalies and shifts in customer arrival times were promptly identified, thereby facilitating proactive management and timely interventions. On the whole, the application of the EWMA function enhanced the analysis and interpretation of arrival time data, leading to improved resource allocation, operational planning, and customer service management within the retail store. Figure 7 displays the inter-arrival time both before and after the application of EWMA at a value of λ = 0.01. The figure clearly illustrates a tendency towards increased symmetry around the mean distribution.

## Conclusions

Monte Carlo simulation methodology was used to generate Gamma distributed data, which then was processes with the EWMA function and assessed the skewness and kurtosis. The general objective of this study is to evaluate the effect of distribution parameters on the performance of the EWMA control chart. In addition, this study focused on the skewness and kurtosis reduction after data processing using the EWMA function. The findings help researchers and practitioners recommend the EWMA and select the appropriate parameters. The results showed that when decreasing the value of λ from 1 to 0.01, the skewness dropped sharply from about 2 to 0.1874758. The lower considered skewed distribution was Gamma (100, β) along with original skewness of 0.1976 (skewness of 0.2 theoretically). The drop in the skewness was slight from 0.197573 at λ = 1.0 to 0.0343 at λ = 0.01. Therefore, the skewness reduction with decreasing the value of λ was higher for more skewed distribution. Even though, the skewness at λ = 0.01 for Gamma (1, β) was still at six folds of skewness of Gamma (100, β) after processing. The same notices about the trends and relationship discussed in the skewness were exactly found in the kurtosis case. The only difference that was found was that the increment of the curve started linear, then become convex, while the deflection curves started to be convex from the beginning.

This research explores the influence of distribution parameters on the Exponentially Weighted Moving Average (EWMA) control chart's performance. The investigation is rooted in various fundamental presumptions. These include the notion that the data utilized in the study adheres to a Gamma distribution, the Monte Carlo simulation methodology accurately reflects real-world conditions, the EWMA control chart is a suitable instrument for gauging the system under study's performance, and the skewness and kurtosis metrics offer substantial insights into the data's behavior. The research discovered that the EWMA control chart's performance is indeed impacted by the distribution parameters. Specifically, the Gamma distribution's shape parameter notably influences the control chart's competency in detecting shifts in the process mean. Additionally, the study ascertained that skewness and kurtosis metrics can be employed to gather insights into the data's behavior and the EWMA control chart's performance.

Nonetheless, the research has a few limitations. It relies on simulated data and doesn't account for potential confounding variables. Future research trajectories involve investigating real-world data, examining the EWMA control chart's performance under different distributional premises and parameter combinations, contrasting the performance of alternative control charts or statistical methodologies with the EWMA control chart, and considering additional elements or covariates that could influence the correlation between distribution parameters and control chart performance.

In conclusion, this research offers significant insights into how distribution parameters impact the performance of the EWMA control chart. Still, there are several limitations that need consideration when interpreting the results and applying them to practical scenarios. Further research is warranted to address these limitations and deepen our comprehension of the subject matter.

Future research is needed to address these limitations and further our understanding of the topic. Future research could also investigate the development of new statistical techniques specifically designed for neutrosophic data analysis. This would contribute to the advancement of neutrosophic statistics as a field and enhance its applicability in various domains. By exploring the potential integration of neutrosophic statistics in the proposed study and citing relevant literature, researchers can lay the groundwork for future investigations in this promising area.

### Supplementary Information

Supplementary Information 1.

Supplementary Information 2.

Supplementary Information 3.

## Data Availability

All data generated or analyzed during this study are included in this published article and its supplementary information files and additional data are available from the corresponding author on reasonable request.
