# Supplementary material for: Symmetry of gamma distribution data about the mean after processing with EWMA function
Source: Sci Rep. 2023 Sep 12;13:15096. doi: 10.1038/s41598-023-39763-6 (PMC10497503; doi:10.1038/s41598-023-39763-6)
Supplement: Supplementary file 1 — Supplementary Information 1. [file 41598_2023_39763_MOESM1_ESM.docx]

Appendix 1. Comparison between the theoretical and simulation results for the mean, variance and skewness of the generated random variates.

| λ | α | β | Mean (Theoretical) | Mean (Simulation) | Error (Fraction) | Variance (Theoretical) | Variance (Simulation) | Error (Fraction) | Skewness (Theoretical) | Skewness (Simulation) | Error (Fraction) |
| --- | --- | --- | --- | --- | --- | --- | --- | --- | --- | --- | --- |
| 0.01 | 1 | 1 | 1 | 0.9984086 | 0.0015914 | 1 | 0.9985655 | 0.0014366 | 2 | 2.0106209 | 0.0053105 |
| 0.05 | 1 | 1 | 1 | 1.0012669 | 0.0012669 | 1 | 0.999311 | 0.0006894 | 2 | 1.9888728 | 0.0055636 |
| 0.1 | 1 | 1 | 1 | 1.0001877 | 0.0001877 | 1 | 1.0024668 | 0.0024608 | 2 | 2.0053914 | 0.0026957 |
| 0.2 | 1 | 1 | 1 | 1.0007807 | 0.0007807 | 1 | 1.0014799 | 0.0014777 | 2 | 2.0015537 | 0.0007768 |
| 0.3 | 1 | 1 | 1 | 0.9995569 | 0.0004431 | 1 | 1.0026206 | 0.0026137 | 2 | 2.0173824 | 0.0086912 |
| 0.4 | 1 | 1 | 1 | 0.9979295 | 0.0020705 | 1 | 0.9931222 | 0.0069254 | 2 | 1.9906861 | 0.0046569 |
| 0.5 | 1 | 1 | 1 | 1.0000643 | 6.431E-05 | 1 | 0.9969419 | 0.0030675 | 2 | 1.9846242 | 0.0076879 |
| 0.6 | 1 | 1 | 1 | 1.0009416 | 0.0009416 | 1 | 1.0048067 | 0.0047837 | 2 | 2.0229786 | 0.0114893 |
| 0.7 | 1 | 1 | 1 | 1.0003837 | 0.0003837 | 1 | 0.9970841 | 0.0029245 | 2 | 1.9926329 | 0.0036836 |
| 0.8 | 1 | 1 | 1 | 1.0004448 | 0.0004448 | 1 | 1.0017828 | 0.0017797 | 2 | 2.0010723 | 0.0005362 |
| 0.9 | 1 | 1 | 1 | 0.9990808 | 0.0009192 | 1 | 0.9983969 | 0.0016057 | 2 | 2.0017296 | 0.0008648 |
| 1 | 1 | 1 | 1 | 0.9991084 | 0.0008916 | 1 | 0.9976476 | 0.002358 | 2 | 1.9997774 | 0.0001113 |
| 0.01 | 2 | 1 | 2 | 2.0003979 | 0.0001989 | 2 | 1.9990735 | 0.0004635 | 1.4142136 | 1.40931 | 0.0034674 |
| 0.05 | 2 | 1 | 2 | 1.9975791 | 0.0012105 | 2 | 1.999552 | 0.000224 | 1.4142136 | 1.4098265 | 0.0031021 |
| 0.1 | 2 | 1 | 2 | 2.0012812 | 0.0006406 | 2 | 2.0079814 | 0.0039748 | 1.4142136 | 1.4141076 | 7.496E-05 |
| 0.2 | 2 | 1 | 2 | 1.9988773 | 0.0005614 | 2 | 2.0000202 | 1.01E-05 | 1.4142136 | 1.4125456 | 0.0011795 |
| 0.3 | 2 | 1 | 2 | 1.9993096 | 0.0003452 | 2 | 1.9935955 | 0.0032125 | 1.4142136 | 1.4021478 | 0.0085318 |
| 0.4 | 2 | 1 | 2 | 2.0018158 | 0.0009079 | 2 | 2.004941 | 0.0024644 | 1.4142136 | 1.4131729 | 0.0007359 |
| 0.5 | 2 | 1 | 2 | 2.0010659 | 0.000533 | 2 | 2.005184 | 0.0025853 | 1.4142136 | 1.4262762 | 0.0085296 |
| 0.6 | 2 | 1 | 2 | 2.0007677 | 0.0003838 | 2 | 2.0059373 | 0.0029599 | 1.4142136 | 1.4210897 | 0.0048622 |
| 0.7 | 2 | 1 | 2 | 2.0004304 | 0.0002152 | 2 | 1.9989818 | 0.0005094 | 1.4142136 | 1.4113202 | 0.0020459 |
| 0.8 | 2 | 1 | 2 | 1.9986981 | 0.0006509 | 2 | 1.9965448 | 0.0017306 | 1.4142136 | 1.4122883 | 0.0013614 |
| 0.9 | 2 | 1 | 2 | 2.0012875 | 0.0006438 | 2 | 2.0004718 | 0.0002359 | 1.4142136 | 1.4029414 | 0.0079706 |
| 1 | 2 | 1 | 2 | 1.9988349 | 0.0005826 | 2 | 1.9968541 | 0.0015754 | 1.4142136 | 1.4153037 | 0.0007709 |
| 0.01 | 3 | 1 | 3 | 3.0016326 | 0.0005442 | 3 | 3.0057174 | 0.0019022 | 1.1547005 | 1.1595789 | 0.0042248 |
| 0.05 | 3 | 1 | 3 | 3.0014989 | 0.0004996 | 3 | 3.0056011 | 0.0018635 | 1.1547005 | 1.1447473 | 0.0086198 |
| 0.1 | 3 | 1 | 3 | 3.0000967 | 3.223E-05 | 3 | 3.0077279 | 0.0025693 | 1.1547005 | 1.1586544 | 0.0034241 |
| 0.2 | 3 | 1 | 3 | 3.0025629 | 0.0008543 | 3 | 2.9936307 | 0.0021276 | 1.1547005 | 1.1463689 | 0.0072155 |
| 0.3 | 3 | 1 | 3 | 2.9995428 | 0.0001524 | 3 | 2.9981627 | 0.0006128 | 1.1547005 | 1.1554716 | 0.0006678 |
| 0.4 | 3 | 1 | 3 | 2.9999285 | 2.383E-05 | 3 | 2.9941432 | 0.0019561 | 1.1547005 | 1.1522983 | 0.0020804 |
| 0.5 | 3 | 1 | 3 | 3.0020853 | 0.0006951 | 3 | 3.002176 | 0.0007248 | 1.1547005 | 1.1572698 | 0.002225 |
| 0.6 | 3 | 1 | 3 | 3.0025578 | 0.0008526 | 3 | 3.005159 | 0.0017167 | 1.1547005 | 1.1514081 | 0.0028513 |
| 0.7 | 3 | 1 | 3 | 2.9988454 | 0.0003849 | 3 | 2.9989173 | 0.000361 | 1.1547005 | 1.1568679 | 0.001877 |
| 0.8 | 3 | 1 | 3 | 3.0010363 | 0.0003454 | 3 | 3.0098152 | 0.0032611 | 1.1547005 | 1.1579198 | 0.0027879 |
| 0.9 | 3 | 1 | 3 | 2.9979467 | 0.0006844 | 3 | 2.9919632 | 0.0026861 | 1.1547005 | 1.1412089 | 0.0116841 |
| 1 | 3 | 1 | 3 | 3.0003545 | 0.0001182 | 3 | 2.9913185 | 0.0029022 | 1.1547005 | 1.1516913 | 0.0026061 |
| 0.01 | 4 | 1 | 4 | 3.9998514 | 3.715E-05 | 4 | 3.9975511 | 0.0006126 | 1 | 1.0030157 | 0.0030157 |
| 0.05 | 4 | 1 | 4 | 3.99965 | 8.749E-05 | 4 | 3.995959 | 0.0010113 | 1 | 1.0034308 | 0.0034308 |
| 0.1 | 4 | 1 | 4 | 4.002498 | 0.0006245 | 4 | 4.0167855 | 0.0041788 | 1 | 1.0089914 | 0.0089914 |
| 0.2 | 4 | 1 | 4 | 4.001369 | 0.0003423 | 4 | 3.9932762 | 0.0016838 | 1 | 0.9926533 | 0.0073467 |
| 0.3 | 4 | 1 | 4 | 4.0009609 | 0.0002402 | 4 | 4.0087097 | 0.0021727 | 1 | 1.0043293 | 0.0043293 |
| 0.4 | 4 | 1 | 4 | 4.0026027 | 0.0006507 | 4 | 4.0000135 | 3.367E-06 | 1 | 0.9991487 | 0.0008513 |
| 0.5 | 4 | 1 | 4 | 3.9971215 | 0.0007196 | 4 | 4.0079161 | 0.0019751 | 1 | 1.0046859 | 0.0046859 |
| 0.6 | 4 | 1 | 4 | 3.9982749 | 0.0004313 | 4 | 3.9973596 | 0.0006605 | 1 | 1.0038639 | 0.0038639 |
| 0.7 | 4 | 1 | 4 | 4.0028404 | 0.0007101 | 4 | 4.008464 | 0.0021115 | 1 | 1.0011371 | 0.0011371 |
| 0.8 | 4 | 1 | 4 | 3.9966841 | 0.000829 | 4 | 3.9978314 | 0.0005424 | 1 | 0.997187 | 0.002813 |
| 0.9 | 4 | 1 | 4 | 3.9985441 | 0.000364 | 4 | 3.9979431 | 0.0005145 | 1 | 0.999409 | 0.000591 |
| 1 | 4 | 1 | 4 | 4.0005357 | 0.0001339 | 4 | 3.9987374 | 0.0003158 | 1 | 0.9990683 | 0.0009317 |
| 0.01 | 5 | 1 | 5 | 5.0011929 | 0.0002386 | 5 | 5.0039015 | 0.0007797 | 0.8944272 | 0.8916788 | 0.0030728 |
| 0.05 | 5 | 1 | 5 | 5.0015168 | 0.0003034 | 5 | 4.9911912 | 0.0017649 | 0.8944272 | 0.8950722 | 0.0007212 |
| 0.1 | 5 | 1 | 5 | 4.9985849 | 0.000283 | 5 | 5.0066679 | 0.0013318 | 0.8944272 | 0.8938904 | 0.0006002 |
| 0.2 | 5 | 1 | 5 | 5.0015543 | 0.0003109 | 5 | 5.0146544 | 0.0029223 | 0.8944272 | 0.8958729 | 0.0016164 |
| 0.3 | 5 | 1 | 5 | 4.9967142 | 0.0006572 | 5 | 4.9846928 | 0.0030708 | 0.8944272 | 0.8951646 | 0.0008244 |
| 0.4 | 5 | 1 | 5 | 5.0021023 | 0.0004205 | 5 | 5.0002272 | 4.544E-05 | 0.8944272 | 0.8996978 | 0.0058927 |
| 0.5 | 5 | 1 | 5 | 5.0034088 | 0.0006818 | 5 | 5.0073831 | 0.0014744 | 0.8944272 | 0.8931187 | 0.001463 |
| 0.6 | 5 | 1 | 5 | 4.996688 | 0.0006624 | 5 | 4.9769129 | 0.0046388 | 0.8944272 | 0.8904185 | 0.0044819 |
| 0.7 | 5 | 1 | 5 | 4.9981091 | 0.0003782 | 5 | 4.984515 | 0.0031066 | 0.8944272 | 0.8954544 | 0.0011484 |
| 0.8 | 5 | 1 | 5 | 4.9998048 | 3.904E-05 | 5 | 5.0004767 | 9.533E-05 | 0.8944272 | 0.8926879 | 0.0019446 |
| 0.9 | 5 | 1 | 5 | 5.002327 | 0.0004654 | 5 | 5.0111101 | 0.0022171 | 0.8944272 | 0.9031243 | 0.0097237 |
| 1 | 5 | 1 | 5 | 5.0004872 | 9.745E-05 | 5 | 5.0053612 | 0.0010711 | 0.8944272 | 0.8939942 | 0.0004841 |
| 0.01 | 6 | 1 | 6 | 6.0024696 | 0.0004116 | 6 | 6.0110083 | 0.0018314 | 0.8164966 | 0.8184253 | 0.0023622 |
| 0.05 | 6 | 1 | 6 | 5.9996468 | 5.886E-05 | 6 | 6.0105815 | 0.0017605 | 0.8164966 | 0.8211282 | 0.0056726 |
| 0.1 | 6 | 1 | 6 | 5.9969738 | 0.0005044 | 6 | 5.9887107 | 0.0018851 | 0.8164966 | 0.8162543 | 0.0002967 |
| 0.2 | 6 | 1 | 6 | 5.9986646 | 0.0002226 | 6 | 5.9932252 | 0.0011304 | 0.8164966 | 0.8172378 | 0.0009078 |
| 0.3 | 6 | 1 | 6 | 6.001383 | 0.0002305 | 6 | 6.0090163 | 0.0015005 | 0.8164966 | 0.8150054 | 0.0018263 |
| 0.4 | 6 | 1 | 6 | 5.9993017 | 0.0001164 | 6 | 5.9971711 | 0.0004717 | 0.8164966 | 0.8171875 | 0.0008462 |
| 0.5 | 6 | 1 | 6 | 5.9980914 | 0.0003181 | 6 | 5.9869418 | 0.0021811 | 0.8164966 | 0.8149517 | 0.0018921 |
| 0.6 | 6 | 1 | 6 | 6.0074047 | 0.0012341 | 6 | 6.0148866 | 0.002475 | 0.8164966 | 0.8175512 | 0.0012916 |
| 0.7 | 6 | 1 | 6 | 6.0013375 | 0.0002229 | 6 | 6.0137519 | 0.0022867 | 0.8164966 | 0.8166913 | 0.0002385 |
| 0.8 | 6 | 1 | 6 | 6.0009222 | 0.0001537 | 6 | 6.0033694 | 0.0005613 | 0.8164966 | 0.8149682 | 0.0018719 |
| 0.9 | 6 | 1 | 6 | 5.9962638 | 0.0006227 | 6 | 5.9989788 | 0.0001702 | 0.8164966 | 0.8130985 | 0.0041618 |
| 1 | 6 | 1 | 6 | 6.0017263 | 0.0002877 | 6 | 5.9886006 | 0.0019035 | 0.8164966 | 0.8109329 | 0.0068141 |
| 0.01 | 7 | 1 | 7 | 6.9981216 | 0.0002683 | 7 | 6.9923897 | 0.0010884 | 0.7559289 | 0.7555677 | 0.0004779 |
| 0.05 | 7 | 1 | 7 | 6.9984887 | 0.0002159 | 7 | 6.9805007 | 0.0027934 | 0.7559289 | 0.7495829 | 0.0083951 |
| 0.1 | 7 | 1 | 7 | 7.0001041 | 1.488E-05 | 7 | 6.9895397 | 0.0014966 | 0.7559289 | 0.7523656 | 0.0047139 |
| 0.2 | 7 | 1 | 7 | 6.9984971 | 0.0002147 | 7 | 7.0140524 | 0.0020035 | 0.7559289 | 0.7604167 | 0.0059367 |
| 0.3 | 7 | 1 | 7 | 6.9991203 | 0.0001257 | 7 | 6.9891096 | 0.0015582 | 0.7559289 | 0.7524726 | 0.0045723 |
| 0.4 | 7 | 1 | 7 | 7.0033809 | 0.000483 | 7 | 7.0028894 | 0.0004126 | 0.7559289 | 0.7526923 | 0.0042817 |
| 0.5 | 7 | 1 | 7 | 7.0001702 | 2.431E-05 | 7 | 7.0003682 | 5.26E-05 | 0.7559289 | 0.7525348 | 0.0044901 |
| 0.6 | 7 | 1 | 7 | 7.0034272 | 0.0004896 | 7 | 7.0123398 | 0.0017597 | 0.7559289 | 0.7611325 | 0.0068837 |
| 0.7 | 7 | 1 | 7 | 6.9957481 | 0.0006074 | 7 | 6.9987268 | 0.0001819 | 0.7559289 | 0.7554413 | 0.0006451 |
| 0.8 | 7 | 1 | 7 | 7.004028 | 0.0005754 | 7 | 7.0107696 | 0.0015362 | 0.7559289 | 0.7572606 | 0.0017616 |
| 0.9 | 7 | 1 | 7 | 6.9969273 | 0.000439 | 7 | 6.9773677 | 0.0032437 | 0.7559289 | 0.754328 | 0.0021179 |
| 1 | 7 | 1 | 7 | 6.9988129 | 0.0001696 | 7 | 7.0084285 | 0.0012026 | 0.7559289 | 0.7545028 | 0.0018866 |
| 0.01 | 8 | 1 | 8 | 7.9967399 | 0.0004075 | 8 | 7.991518 | 0.0010614 | 0.7071068 | 0.7048027 | 0.0032584 |
| 0.05 | 8 | 1 | 8 | 7.9993353 | 8.308E-05 | 8 | 8.0102681 | 0.0012819 | 0.7071068 | 0.7094612 | 0.0033296 |
| 0.1 | 8 | 1 | 8 | 8.003305 | 0.0004131 | 8 | 8.0118788 | 0.0014826 | 0.7071068 | 0.7044406 | 0.0037705 |
| 0.2 | 8 | 1 | 8 | 7.9945953 | 0.0006756 | 8 | 7.9901931 | 0.0012274 | 0.7071068 | 0.7065732 | 0.0007546 |
| 0.3 | 8 | 1 | 8 | 8.0000756 | 9.455E-06 | 8 | 8.0255516 | 0.0031838 | 0.7071068 | 0.7111265 | 0.0056847 |
| 0.4 | 8 | 1 | 8 | 8.0013349 | 0.0001669 | 8 | 8.0017067 | 0.0002133 | 0.7071068 | 0.7061627 | 0.0013352 |
| 0.5 | 8 | 1 | 8 | 8.00394 | 0.0004925 | 8 | 7.9847472 | 0.0019102 | 0.7071068 | 0.7038597 | 0.0045921 |
| 0.6 | 8 | 1 | 8 | 7.9994084 | 7.395E-05 | 8 | 8.0175885 | 0.0021937 | 0.7071068 | 0.7091556 | 0.0028975 |
| 0.7 | 8 | 1 | 8 | 8.0024937 | 0.0003117 | 8 | 8.0178156 | 0.002222 | 0.7071068 | 0.7108174 | 0.0052475 |
| 0.8 | 8 | 1 | 8 | 7.9994548 | 6.815E-05 | 8 | 7.9938039 | 0.0007751 | 0.7071068 | 0.7087726 | 0.0023558 |
| 0.9 | 8 | 1 | 8 | 7.9997551 | 3.061E-05 | 8 | 7.9924328 | 0.0009468 | 0.7071068 | 0.704246 | 0.0040458 |
| 1 | 8 | 1 | 8 | 7.9969297 | 0.0003838 | 8 | 8.0069272 | 0.0008651 | 0.7071068 | 0.7076692 | 0.0007953 |
| 0.01 | 9 | 1 | 9 | 8.9969675 | 0.0003369 | 9 | 9.0072992 | 0.0008104 | 0.6666667 | 0.6711642 | 0.0067463 |
| 0.05 | 9 | 1 | 9 | 8.9992732 | 8.076E-05 | 9 | 9.021543 | 0.002388 | 0.6666667 | 0.6683872 | 0.0025807 |
| 0.1 | 9 | 1 | 9 | 8.9972511 | 0.0003054 | 9 | 9.000276 | 3.066E-05 | 0.6666667 | 0.6669336 | 0.0004004 |
| 0.2 | 9 | 1 | 9 | 8.9979674 | 0.0002258 | 9 | 8.9941008 | 0.0006559 | 0.6666667 | 0.6682378 | 0.0023568 |
| 0.3 | 9 | 1 | 9 | 8.9974796 | 0.00028 | 9 | 8.9885813 | 0.0012704 | 0.6666667 | 0.6676516 | 0.0014774 |
| 0.4 | 9 | 1 | 9 | 8.9966949 | 0.0003672 | 9 | 8.9987504 | 0.0001389 | 0.6666667 | 0.6746184 | 0.0119276 |
| 0.5 | 9 | 1 | 9 | 9.0014961 | 0.0001662 | 9 | 8.9978764 | 0.000236 | 0.6666667 | 0.665873 | 0.0011905 |
| 0.6 | 9 | 1 | 9 | 9.0011468 | 0.0001274 | 9 | 8.9893834 | 0.001181 | 0.6666667 | 0.6639776 | 0.0040336 |
| 0.7 | 9 | 1 | 9 | 8.9982524 | 0.0001942 | 9 | 8.9864217 | 0.001511 | 0.6666667 | 0.6649748 | 0.0025378 |
| 0.8 | 9 | 1 | 9 | 8.9969001 | 0.0003444 | 9 | 8.9970079 | 0.0003326 | 0.6666667 | 0.6708695 | 0.0063042 |
| 0.9 | 9 | 1 | 9 | 9.0056079 | 0.0006231 | 9 | 9.0223383 | 0.0024759 | 0.6666667 | 0.6693407 | 0.0040111 |
| 1 | 9 | 1 | 9 | 9.0040513 | 0.0004501 | 9 | 9.0001193 | 1.325E-05 | 0.6666667 | 0.6607028 | 0.0089458 |
| 0.01 | 10 | 1 | 10 | 10.000924 | 9.237E-05 | 10 | 10.008864 | 0.0008856 | 0.6324555 | 0.6304791 | 0.0031251 |
| 0.05 | 10 | 1 | 10 | 10.002497 | 0.0002497 | 10 | 10.002552 | 0.0002551 | 0.6324555 | 0.6299936 | 0.0038926 |
| 0.1 | 10 | 1 | 10 | 10.00511 | 0.000511 | 10 | 9.9931655 | 0.0006839 | 0.6324555 | 0.6323221 | 0.0002109 |
| 0.2 | 10 | 1 | 10 | 9.9983379 | 0.0001662 | 10 | 10.015553 | 0.0015529 | 0.6324555 | 0.6334659 | 0.0015976 |
| 0.3 | 10 | 1 | 10 | 9.994876 | 0.0005124 | 10 | 10.009253 | 0.0009244 | 0.6324555 | 0.6354141 | 0.0046779 |
| 0.4 | 10 | 1 | 10 | 10.003887 | 0.0003887 | 10 | 10.005431 | 0.0005428 | 0.6324555 | 0.6302399 | 0.0035032 |
| 0.5 | 10 | 1 | 10 | 10.007641 | 0.0007641 | 10 | 10.001312 | 0.0001312 | 0.6324555 | 0.6331429 | 0.0010869 |
| 0.6 | 10 | 1 | 10 | 9.9946527 | 0.0005347 | 10 | 10.006789 | 0.0006785 | 0.6324555 | 0.629238 | 0.0050874 |
| 0.7 | 10 | 1 | 10 | 10.001209 | 0.0001209 | 10 | 9.9876972 | 0.0012318 | 0.6324555 | 0.6329376 | 0.0007622 |
| 0.8 | 10 | 1 | 10 | 9.9956312 | 0.0004369 | 10 | 10.021137 | 0.0021092 | 0.6324555 | 0.640012 | 0.0119479 |
| 0.9 | 10 | 1 | 10 | 10.003278 | 0.0003278 | 10 | 10.03449 | 0.0034372 | 0.6324555 | 0.6307575 | 0.0026848 |
| 1 | 10 | 1 | 10 | 9.9914373 | 0.0008563 | 10 | 9.9647235 | 0.0035401 | 0.6324555 | 0.6325909 | 0.0002141 |
| 0.01 | 50 | 1 | 50 | 49.989802 | 0.000204 | 50 | 49.949453 | 0.001012 | 0.2828427 | 0.282628 | 0.0007593 |
| 0.05 | 50 | 1 | 50 | 49.997457 | 5.086E-05 | 50 | 50.012832 | 0.0002566 | 0.2828427 | 0.2812891 | 0.0054928 |
| 0.1 | 50 | 1 | 50 | 50.000078 | 1.556E-06 | 50 | 50.01793 | 0.0003585 | 0.2828427 | 0.2793406 | 0.012382 |
| 0.2 | 50 | 1 | 50 | 50.012596 | 0.0002519 | 50 | 49.985812 | 0.0002838 | 0.2828427 | 0.2814936 | 0.0047699 |
| 0.3 | 50 | 1 | 50 | 50.004276 | 8.553E-05 | 50 | 50.014794 | 0.0002958 | 0.2828427 | 0.2817423 | 0.0038907 |
| 0.4 | 50 | 1 | 50 | 50.004425 | 8.85E-05 | 50 | 49.966556 | 0.0006693 | 0.2828427 | 0.2889064 | 0.0214383 |
| 0.5 | 50 | 1 | 50 | 49.994513 | 0.0001097 | 50 | 49.92839 | 0.0014343 | 0.2828427 | 0.2845665 | 0.0060947 |
| 0.6 | 50 | 1 | 50 | 49.995991 | 8.019E-05 | 50 | 50.017439 | 0.0003487 | 0.2828427 | 0.2879504 | 0.0180583 |
| 0.7 | 50 | 1 | 50 | 50.011181 | 0.0002236 | 50 | 50.021805 | 0.0004359 | 0.2828427 | 0.2845584 | 0.0060657 |
| 0.8 | 50 | 1 | 50 | 50.008305 | 0.0001661 | 50 | 50.194473 | 0.0038744 | 0.2828427 | 0.2772601 | 0.0197374 |
| 0.9 | 50 | 1 | 50 | 50.008218 | 0.0001644 | 50 | 49.999294 | 1.412E-05 | 0.2828427 | 0.280274 | 0.0090819 |
| 1 | 50 | 1 | 50 | 50.005374 | 0.0001075 | 50 | 50.009803 | 0.000196 | 0.2828427 | 0.2803671 | 0.0087525 |
| 0.01 | 100 | 1 | 100 | 99.99217 | 7.83E-05 | 100 | 99.904691 | 0.000954 | 0.2 | 0.1975075 | 0.0124625 |
| 0.05 | 100 | 1 | 100 | 99.989329 | 0.0001067 | 100 | 99.810234 | 0.0019013 | 0.2 | 0.1979126 | 0.010437 |
| 0.1 | 100 | 1 | 100 | 99.999495 | 5.048E-06 | 100 | 100.0375 | 0.0003748 | 0.2 | 0.1986064 | 0.0069679 |
| 0.2 | 100 | 1 | 100 | 99.995808 | 4.192E-05 | 100 | 99.843694 | 0.0015655 | 0.2 | 0.1999841 | 7.961E-05 |
| 0.3 | 100 | 1 | 100 | 100.02068 | 0.0002068 | 100 | 99.949829 | 0.000502 | 0.2 | 0.2010018 | 0.0050089 |
| 0.4 | 100 | 1 | 100 | 100.01067 | 0.0001067 | 100 | 99.819173 | 0.0018115 | 0.2 | 0.2034221 | 0.0171105 |
| 0.5 | 100 | 1 | 100 | 99.99284 | 7.16E-05 | 100 | 100.04303 | 0.0004302 | 0.2 | 0.1964669 | 0.0176655 |
| 0.6 | 100 | 1 | 100 | 100.0014 | 1.403E-05 | 100 | 100.1838 | 0.0018347 | 0.2 | 0.2025265 | 0.0126325 |
| 0.7 | 100 | 1 | 100 | 99.997765 | 2.235E-05 | 100 | 100.11185 | 0.0011172 | 0.2 | 0.1989111 | 0.0054444 |
| 0.8 | 100 | 1 | 100 | 100.00871 | 8.708E-05 | 100 | 99.883317 | 0.0011682 | 0.2 | 0.1950271 | 0.0248644 |
| 0.9 | 100 | 1 | 100 | 100.0054 | 5.404E-05 | 100 | 100.23936 | 0.0023879 | 0.2 | 0.2032844 | 0.016422 |
| 1 | 100 | 1 | 100 | 100.00176 | 1.756E-05 | 100 | 100.01788 | 0.0001788 | 0.2 | 0.1980521 | 0.0097394 |
