# Supplementary material for: Symmetry of gamma distribution data about the mean after processing with EWMA function
Source: Sci Rep. 2023 Sep 12;13:15096. doi: 10.1038/s41598-023-39763-6 (PMC10497503; doi:10.1038/s41598-023-39763-6)
Supplement: Supplementary file 2 — Supplementary Information 2. [file 41598_2023_39763_MOESM2_ESM.docx]

Appendix 2. MATLAB code to find the skewness and kurtosis of EWMA statistics with input of Gamma generated random numbers.

**Skewness code .. Substitute the wanted value of i, and j (i.e., shape and scale parameters)**

skewness(i,j)= Myfunction(trials, lamda, alpha, beta);

function Skew= Myfunction(trials, lamda, alpha, beta)

B=zeros(trials,1);

B(1,1)= gamrnd(alpha, beta);

for i=2:trials

B(i,1)= gamrnd(alpha, beta)*lamda+B(i-1,1)*(1-lamda);

end

Skew=skewness(B);

end

**Kurtosis code .. Substitute the wanted value of i, and j (i.e., shape and scale parameters)**

kurtosis(i,j)= Mykurtfunction(trials, lamda, alpha, beta);

function Kurt= MyKurtfunction(trials, lamda, alpha, beta)

B=zeros(trials,1);

B(1,1)= gamrnd(alpha, beta);

for i=2:trials

B(i,1)= gamrnd(alpha, beta)*lamda+B(i-1,1)*(1-lamda);

end

kurt=kurtosis (B);

end
