# Supplementary material for: Symmetry of gamma distribution data about the mean after processing with EWMA function
Source: Sci Rep. 2023 Sep 12;13:15096. doi: 10.1038/s41598-023-39763-6 (PMC10497503; doi:10.1038/s41598-023-39763-6)

Appendix 3.

EWMA Skewness of original distribution of Gamm(1,k), Gamma(10, k) and difference between them


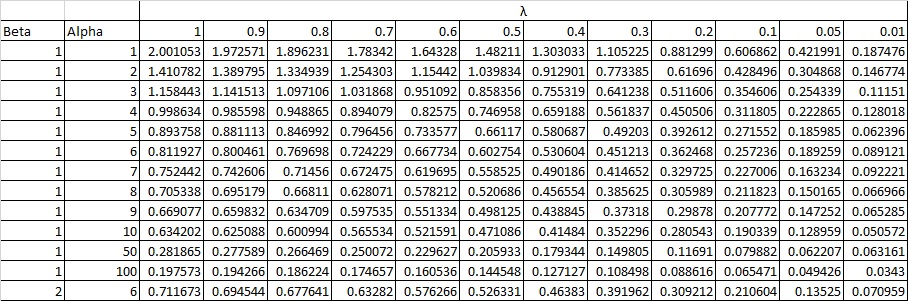

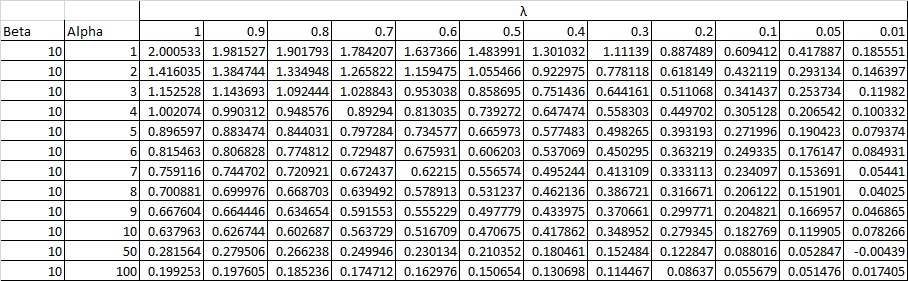

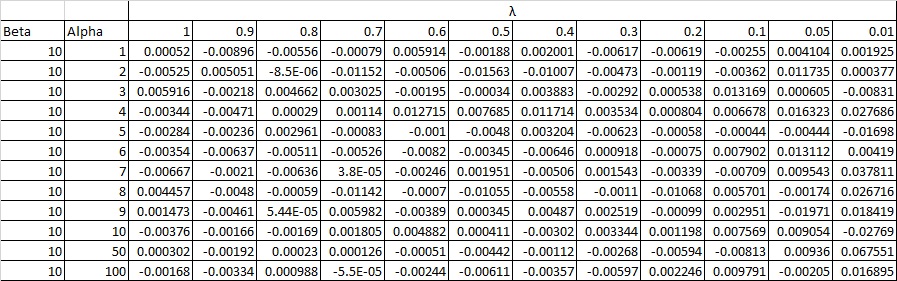


Appendix 3.

EWMA kurtosis of original distribution of Gamm (1, k), Gamma (10, k) and difference between them


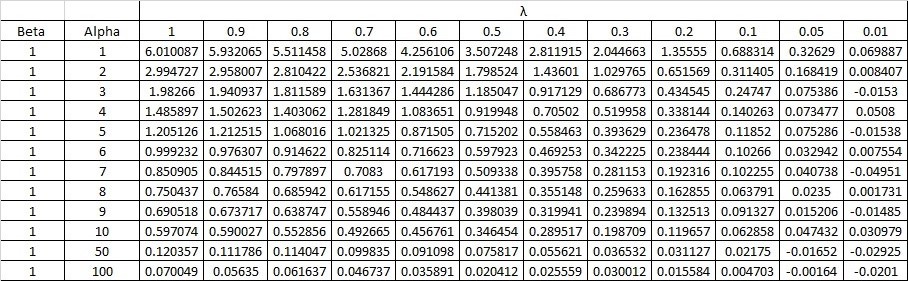

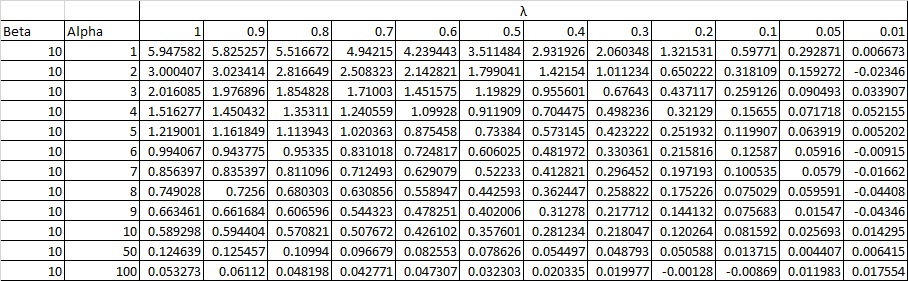

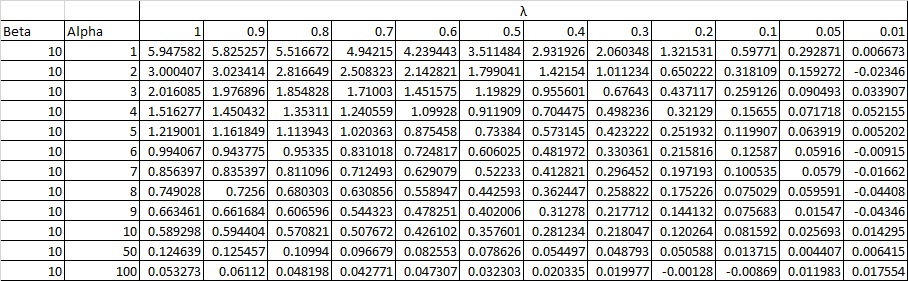

Supplement: Supplementary file 3 — Supplementary Information 3. [file 41598_2023_39763_MOESM3_ESM.docx]
